# Effectiveness and safety of manual therapy for knee osteoarthritis: An overview of systematic reviews and meta-analyses

**DOI:** 10.3389/fpubh.2023.1081238

**Published:** 2023-02-24

**Authors:** Tianxiao Feng, Xu Wang, Zikai Jin, Xiaokuan Qin, Chuanrui Sun, Baoyu Qi, Yili Zhang, Liguo Zhu, Xu Wei

**Affiliations:** ^1^Wangjing Hospital, China Academy of Chinese Medical Sciences, Beijing, China; ^2^School of Traditional Chinese Medicine and School of Integrated Chinese and Western Medicine, Nanjing University of Chinese Medicine, Nanjing, China

**Keywords:** manual therapy, rehabilitation, knee osteoarthritis, systematic review, overview

## Abstract

**Background:**

Manual therapy has been used as an alternative approach to treat knee osteoarthritis (KOA) for many years. Numerous systematic reviews (SRs) or meta-analyses (MAs) were published to evaluate its effectiveness and safety. Nevertheless, the conclusions of SRs/MAs are inconsistent, and the uneven quality needs to be critically appraised.

**Objectives:**

To conduct a comprehensive overview of the effectiveness and safety of manual therapy for KOA and the quality of relevant SRs/MAs, thus providing critical evidence and valuable direction for future researchers to promote the generation of advanced evidence.

**Methods:**

The pre-defined search strategies were applied to eight electronic databases from inception to September 2022. Suitable SRs/MAs were included in accordance with the inclusion and exclusion criteria. The methodological quality, risk of bias, reporting quality, and evidence quality were assessed by two independent reviewers who used respectively the A Measurement Tool to Assess Systematic Reviews 2 (AMSTAR-2), the Risk of Bias in Systematic Reviews (ROBIS), the Preferred Reporting Items for Systematic Reviews and Meta-Analyses 2020 Version (PRISMA 2020), and Grades of Recommendations, Assessment, Development and Evaluation (GRADE) based on the method of narrative synthesis. We excluded the overlapping randomized controlled trials (RCTs) and performed a re-meta-analysis of the total effective rate.

**Results:**

A total of eleven relevant SRs/MAs were included: nine SRs/MAs were rated critically low quality, and two were rated low quality by AMSTAR-2. According to ROBIS, all SRs/MAs were rated low risk in Phase 1 (assessing relevance) and Domain 1 (study eligibility criteria) of Phase 2. Three SRs/MAs (27.27%) were rated low risk in Domain 2 (identification and selection of studies). Ten SRs/MAs (90.91%) were rated low risk in Domain 3 (data collection and study appraisal). Five SRs/MAs (45.45%) were rated low risk in Domain 4 (synthesis and findings). And five SRs/MAs (45.45%) were rated low risk in Phase 3 (risk of bias in the review). By PRISMA 2020, there were some reporting deficiencies in the aspects of abstract (2/11, 18.18%), search strategy (0/11, 0%), preprocessing of merging data (0/11, 0%), heterogeneity exploration (6/11, 54.55%), sensitivity analysis (4/11, 36.36%), publication bias (5/11, 45.45%), evidence quality (3/11, 27.27%), the list of excluded references (3/11, 27.27%), protocol and registration (1/11, 9.09%), funding (1/11, 9.09%), conflict of interest (3/11, 27.27%), and approach to relevant information (0/11, 0%). In GRADE, the evidence quality was defined as moderate quality (8 items, 21.05%), low quality (16 items, 42.11%), and critically low quality (14 items, 36.84%). Among the downgraded factors, risk of bias, inconsistency, imprecision, and publication bias were the main factors. A re-meta-analysis revealed that manual therapy can increase the total effective rate in KOA patients (risk ratio = 1.15, 95% confidence interval [1.12, 1.18], *p* < 0.00001; I^2^ = 0, *p* = 0.84). There are four reviews that narratively report adverse effects, and no severe adverse reactions occurred in the manual therapy group.

**Conclusions:**

Manual therapy may be clinically effective and safe for patients with KOA. However, this conclusion must be interpreted with caution because of the generally unsatisfactory study quality and inconsistent conclusions of the included SRs/MAs. Further rigorous and normative SRs/MAs are expected to be carried out to provide robust evidence for definitive conclusions.

**Systematic review registration:**

https://www.crd.york.ac.uk/PROSPERO/#myprospero, identifier: CRD42022364672.

## 1. Introduction

Knee osteoarthritis (KOA), the most prevalent form of osteoarthritis, is a chronic degenerative joint pathology characterized by progressive hyaline articular cartilage destruction, sclerotic changes of subchondral bone, and synovial inflammation (1, 2). It is a leading cause of disability and poor quality of life worldwide due to the symptoms and signs including chronic knee pain, stiffness, functional limitations, and muscle weakness (3, 4). Nearly 21% of patients undergoing KOA even suffer from a series of psychological problems such as depression and anxiety (5). The global prevalence of KOA has reached 22.9% in patients aged 40 or older, which represents tremendous personal and societal burdens (6, 7). The increased disease burden has not only strained severely the healthcare institution but has also affected the medical expenditure of patients (8). As life expectancy and obesity prevalence increase, the number of people living for prolonged periods with KOA is expected to grow in the foreseeable future (9). Thus, the management of patients with KOA attracts increasing attention from researchers.

At present, the primary treatment goals of KOA are to decrease pain, enhance physical function, and improve the quality of life (10). Doctors need to develop individualized and stepwise treatment strategies based on the actual situations of their patients. Current guidelines have evaluated over 50 treatments for KOA (11–14). Common interventions for KOA include patient education, weight management, exercise therapy, physical therapy, pharmacologic therapy, and surgery therapy (15, 16). Moreover, several innovative therapies for KOA, such as stem cell therapy (17), chondrocyte cell-sheet transplantation (18), injectable natural polymer hydrogels (19), geniculate artery embolization (20), and water-cooled radiofrequency ablation (21), have been proven effective by relevant studies. However, more rigorous clinical trials are needed to determine whether those treatments can be recommended in clinical practice. Drug analgesic intervention is still the primary therapy for KOA. The majority of the physicians generally prescribe medications for controlling the symptoms. Oral medications mainly contain acetaminophen, non-steroidal anti-inflammatory drugs (NSAIDs), opioids, and duloxetine. However, the long-term efficacy and side effects of pharmacotherapy are still uncertain (22). Some rehabilitation strategies for individuals with KOA are more likely to alleviate the pain in the long term and to delay functional decline than the existing drugs (1). And the proportion of patients who have multiple coexisting diseases simultaneously is rising steadily in older people (23, 24). The multimorbidity trend presents challenges to the pharmacotherapy of KOA. For example, in patients with severe gastrointestinal or cardiovascular conditions, the use of oral NSAIDs is not recommended owing to the adverse effects such as gastrointestinal events (irritation, ulceration, or bleeding) and cardiovascular events (myocardial infarct, cerebrovascular accidents, or hypertension) (11, 16). Therefore, many patients have gradually shifted from drug interventions to seeking rehabilitation techniques in recent years (25).

Rehabilitation techniques largely meet a tremendous need for conservative management to enhance physical functioning and quality of life (26). Manual therapy has been used as part of a multimodal rehabilitation management for KOA as it has the potential to improve symptoms. It can be defined as the application of a manual force to the patient by a trained practitioner to improve pain-related symptoms and mobility in areas that are restricted, such as skeletal muscles, joints, connective tissues, or nerve tissues (27). Manual therapy mainly includes soft tissue techniques, stretching, massage, active or passive mobilization, and manipulation techniques (28, 29), which have been popular therapeutic modalities for patients with KOA. Many randomized controlled trials (RCTs) have been conducted to investigate the effect of manual therapy in the treatment of KOA. Pozsgai et al. (30) reported that single manual therapy is effective immediately and in short term on alleviation of pain compared to sham manual therapy. Nigam et al. (31) reported long-term beneficial effects (up to 6 months) of manual therapy, and their results indicated that the addition of manual therapy provided clinically significant improvements in pain, disability, and functional activities than usual care alone in patients with symptomatic KOA. The exact mechanism of manual therapy is unknown, but it is postulated that it may improve blood supply, enhance muscle strength, relieve inflammatory reactions, and also result in changes in the immune system, which may help improve pain and physical function in patients with KOA (32). To date, numerous systematic reviews (SRs) or meta-analyses (MAs) about manual therapy for KOA have been reported. However, the effectiveness and safety of manual therapy from related research still didn't reach a consistent conclusion. Owing to poor data quality and an insufficient benefit, the level of evidence for manual therapy is ambiguous, and many clinical practice guidelines have not yet recommended manual therapy as a routine treatment for KOA in clinical practice. Therefore, credible evidence of manual therapy for KOA is still needed.

**Figure 1:**
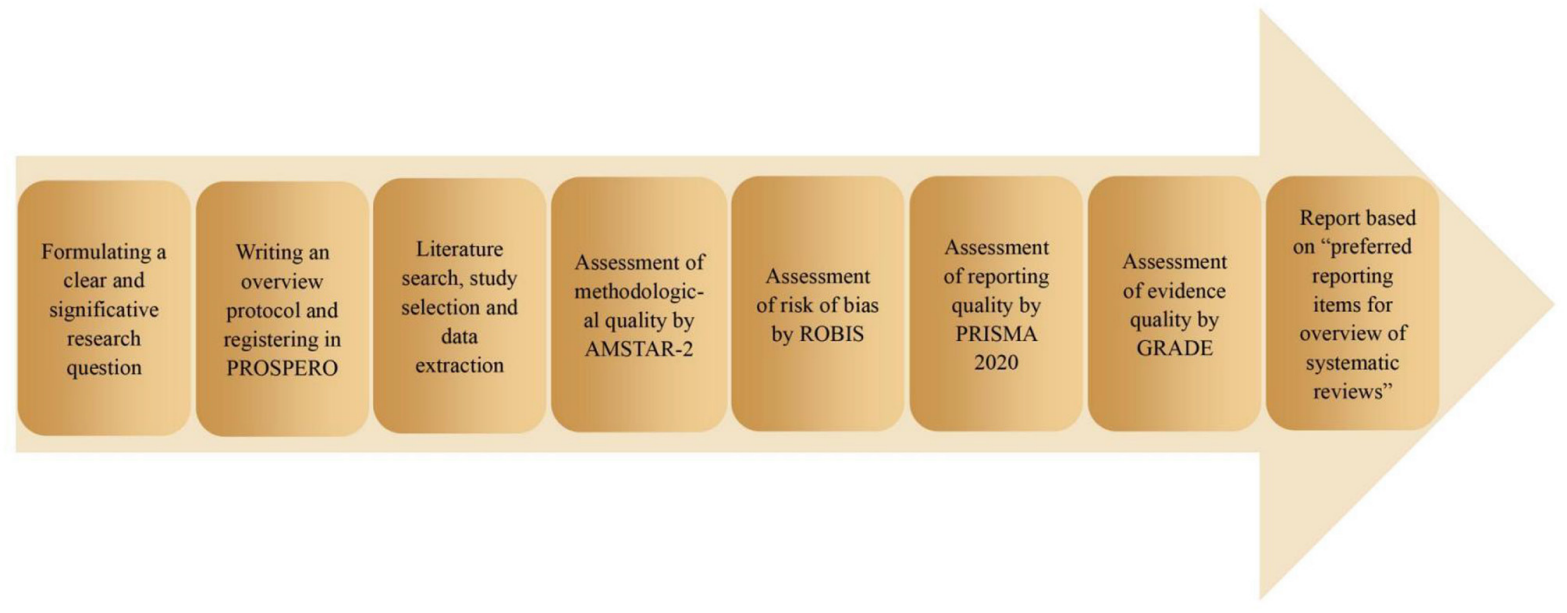
The process of this overview.

An overview is a method of summarizing study evidence from different SRs/MAs into one usable and accessible document (33). It can provide a comprehensive description by assessing the methodological quality, reporting quality, risk of bias, and evidence quality of relevant SRs/MAs. To our knowledge, no overview of manual therapy for KOA has been published. Therefore, we conducted this overview to comprehensively evaluate the effectiveness and safety of manual therapy for KOA based on relevant SRs/MAs (Figure 1). This overview will provide critical evidence for clinicians, patients, or policymakers, as well as improvement guidance for SR/MA producers in the future.

## 2. Methods

### 2.1. Protocol and registration

The protocol of this overview was registered in the International Prospective Register of Systematic Reviews (PROSPERO) (https://www.crd.york.ac.uk/PROSPERO/) with the registration number CRD42022364672.

### 2.2. Search strategy

Two reviewers (TF and ZJ) independently searched the literature in eight electronic databases, including PubMed, EMBASE, Cochrane Library, Web of Science, the Chinese National Knowledge Infrastructure (CNKI), the WanFang Database, Chinese Biological Medicine (CBM), and the Chongqing VIP Database, from inception to September 2022. In addition, relevant references for review articles, the research registries and gray literature such as academic dissertations and conference reports were further searched manually. There were no restrictions on the language or the publication status. The search terms mainly include “Osteoarthritis, Knee”, “Osteoarthritis”, “Knee osteoarthritis”, “Knee pain”, “KOA”, “Manipulation, Osteopathic”, “Massage”, “Chiropractic”, “Manual therapy”, “Manipulation therapy”, “Osteopathic manipulative treatment”, “Meta-analysis”, and “Systematic review”. The search strategies were adjusted to suit the specific features of the electronic databases. The search strategy for PubMed was shown in Table 1. Detailed search strategies for electronic databases were summarized in Appendix A.

**Table 1:** The search strategy for PubMed.

**Query**	**Search term**
#1	Massage [Mesh]
#2	Manipulation, Osteopathic [Mesh]
#3	Chiropractic [Mesh]
#4	Zone Therapy [Title/Abstract] OR Zone Therapies [Title/Abstract] OR Massage Therapy [Title/Abstract] OR Massage Therapies [Title/Abstract] OR Osteopathic Manipulative Treatment [Title/Abstract] OR Osteopathic Manipulative Treatments [Title/Abstract] OR Osteopathic Manipulation [Title/Abstract] OR Tuina [Title/Abstract] OR Manual Therapy [Title/Abstract] OR Manual Traction [Title/Abstract] OR Manipulation Therapy [Title/Abstract] OR Manipulative Therapy [Title/Abstract] OR Massage [Title/Abstract] OR Manipulation, Osteopathic [Title/Abstract] OR Chiropractic [Title/Abstract]
#5	#1 OR #2 OR #3 OR #4
#6	Osteoarthritis, Knee [Mesh]
#7	Osteoarthritis [Mesh]
#8	Knee Osteoarthritides [Title/Abstract] OR Knee Osteoarthritis [Title/Abstract] OR Osteoarthritis of Knee [Title/Abstract] OR Osteoarthritis [Title/Abstract] OR Osteoarthritides [Title/Abstract] OR Osteoarthrosis [Title/Abstract] OR Osteoarthroses [Title/Abstract] OR Arthritis, Degenerative [Title/Abstract] OR Arthritides, Degenerative [Title/Abstract] OR Degenerative Arthritides [Title/Abstract] OR Degenerative Arthritis [Title/Abstract] OR Knee Pain [Title/Abstract] OR KOA [Title/Abstract]
#9	#6 OR #7 OR #8
#10	Systematic reviews as topic [Mesh]
#11	Meta-analysis as topic [Mesh]
#12	Systematic review [Publication Type]
#13	Meta-analysis [Publication Type]
#14	Meta-analysis [Title/Abstract] OR Systematic reviews [Title/Abstract] OR Systematic review [Title/Abstract] OR Meta analysis [Title/Abstract] OR Meta analyses [Title/Abstract] OR Meta-analyses [Title/Abstract] OR Evaluation of system [Title/Abstract] OR System assessment [Title/Abstract] OR System evaluation [Title/Abstract] OR Systematic assessment [Title/Abstract]
#15	#10 OR #11 OR #12 OR #13 OR #14
#16	#5 AND #9 AND #15

### 2.3. Inclusion criteria

#### 2.3.1. Type of studies

SRs/MAs containing more than one RCT that used manual therapy for KOA were eligible.

#### 2.3.2. Type of participants

According to the existing internationally recognized diagnostic criteria, patients who were diagnosed with KOA were included regardless of the differences in gender, region, age, ethnicity, disease duration, or severity.

#### 2.3.3. Type of interventions

Manual therapy was the primary intervention measure, with no restrictions on types of manual therapy (such as massage, joint mobilization, manipulation, or other manual therapies). It could be treated with manual therapy alone or combined with the control intervention.

#### 2.3.4. Type of comparators

The control interventions included sham (placebo) manual therapy, exercise therapy, usual care, western medicine, acupuncture therapy, no treatment, or other conventional treatments.

#### 2.3.5. Type of outcome measures

The primary outcome was the total effective rate. The total effective rate was a compound outcome and total effective rate = (number of basically cured patients + number of markedly improved patients + number of improved patients) / total number of patients (34). The secondary outcomes included the Visual Analog Scale (VAS), Western Ontario and McMaster Universities Osteoarthritis Index (WOMAC) pain score, WOMAC stiffness score, WOMAC physical function score, hospital for special surgery knee score (HSS), and stairs ascending-descending time. These outcome measures have been widely used as evaluation tools by clinical investigators to observe the efficacy of KOA. The VAS score is the most frequently used instrument to assess pain intensity in patients with chronic musculoskeletal pain (35). The WOMAC score is a patient-reported questionnaire that can be used to assess pain, stiffness, and physical function for osteoarthritis of the hip or knee (36). HSS score and stairs ascending-descending time are reliable and effective outcome measures for evaluating the physical function and performance of KOA (37, 38).

### 2.4. Exclusion criteria

The exclusion criteria were as follows: (a) SRs/MAs including non-RCTs; (b) SRs/MAs without quantitative synthesis; (c) The control interventions were treated with different types of manual therapy; (d) Network meta-analyses; (e) Duplicate publication; (f) Protocols of SRs/MAs; (g) SRs/MAs whose full text couldn't be accessed; (h) SRs/MAs without the outcomes mentioned above.

### 2.5. Study selection

According to the intended inclusion and exclusion criteria, two reviewers (TF and XQ) conducted literature screening independently. Two reviewers imported the retrieved results into Endnote X9.3 software to remove duplicates. Inconsistent articles were then excluded based on their titles and abstracts. Finally, eligible SRs/MAs were retrieved for full-text assessment. Any unresolved disagreements were resolved by a third reviewer (XW).

### 2.6. Data extraction

Two reviewers (TF and XQ) independently extracted data by using a pre-designed information extraction table, and the extraction items were as follows: the first author, publication year, country, language, number of included RCTs, total simple size, type of intervention and comparator, outcome measures, duration of treatment, quality assessment tool, data analysis methods, and overall conclusions. Two reviewers cross-checked the extracted content and consulted a third reviewer (XW) for any disagreements.

### 2.7. Quality assessment

The quality assessment of the overview mainly followed the Cochrane Handbook and the methods of relevant studies (39–43). The quality assessment mainly contained four aspects: methodological quality, risk of bias, reporting quality, and evidence quality. Two independent reviewers (TF and XW) evaluated the quality of the SRs/MAs. Before the evaluation, each item of the relevant assessment tools was intensively discussed to reach a consensus.

#### 2.7.1. Assessment of methodological quality

A Measurement Tool to Assess Systematic Reviews 2 (AMSTAR-2) (44) was applied to assess the methodological quality of included SRs/MAs. The AMSTAR-2 contains 16 items (Appendix B), including 7 critical items (items 2, 4, 7, 9, 11, 13, 15). Each item is evaluated as “yes”, “partial yes”, or “no” according to the standard. An overall assessment of SRs/MAs (high, medium, low, or critically low) is performed based on the evaluation of critical and non-critical items.

#### 2.7.2. Assessment of risk of bias

Risk of Bias in Systematic Reviews (ROBIS) (45) was applied to assess the risk of bias based on 3 phases (Appendix C). Phase 1 assesses whether the proposed question matches the target question from participants, interventions, comparisons, and outcomes (PICO). Phase 2 consists of 4 domains: “study eligibility criteria”, “identification and selection of studies”, “data collection and study appraisal”, and “synthesis and findings”. Phase 3 is based on the evaluation of phase 2 domains for comprehensive assessment. The risk of bias of SRs/MAs is evaluated as “low risk”, “high risk” or “unclear risk”.

#### 2.7.3. Assessment of reporting quality

Preferred Reporting Items for Systematic Reviews and Meta-Analyses 2020 Version (PRISMA 2020) (46) was applied to assess the reporting quality of included SRs/MAs. The PRISMA 2020 consists of 27 items (Appendix D), covering seven aspects of SRs/MAs, including the title, abstract, introduction, methods, results, discussion, and other information. Each item is evaluated as “yes”, “partial yes”, or “no”.

#### 2.7.4. Assessment of evidence quality

The Grades of Recommendations, Assessment, Development and Evaluation (GRADE) (47) system was applied to assess the quality of the evidence. Evidence based on RCTs begins as high quality, but confidence may decrease according to five aspects: risk of bias, inconsistency, indirectness, imprecision, and publication bias. The quality of the evidence is graded “high”, “moderate”, “low”, or “critically low” (Appendix E).

### 2.8. Data synthesis and analysis

The clinical characteristics of reviews and the results of AMSTAR-2, ROBIS, PRISMA 2020, and GRADE were summarized by tables or figures based on the method of narrative synthesis. GRADE profiler 3.6.1 software played an important role in assessing the evidence quality. Review Manager 5.4 software was used in the re-meta-analysis of the primary outcome. A dichotomous variable was represented by the risk ratio (RR) and 95% confidence interval (CI). When there is obvious heterogeneity (I^2^ > 50%), the random-effects model should be applied. When no significant heterogeneity (I^2^ < 50%), the fixed-effects model was used. A funnel plot was used to detect publication bias.

## 3. Results

### 3.1. Study identification and selection

**Figure 2:**
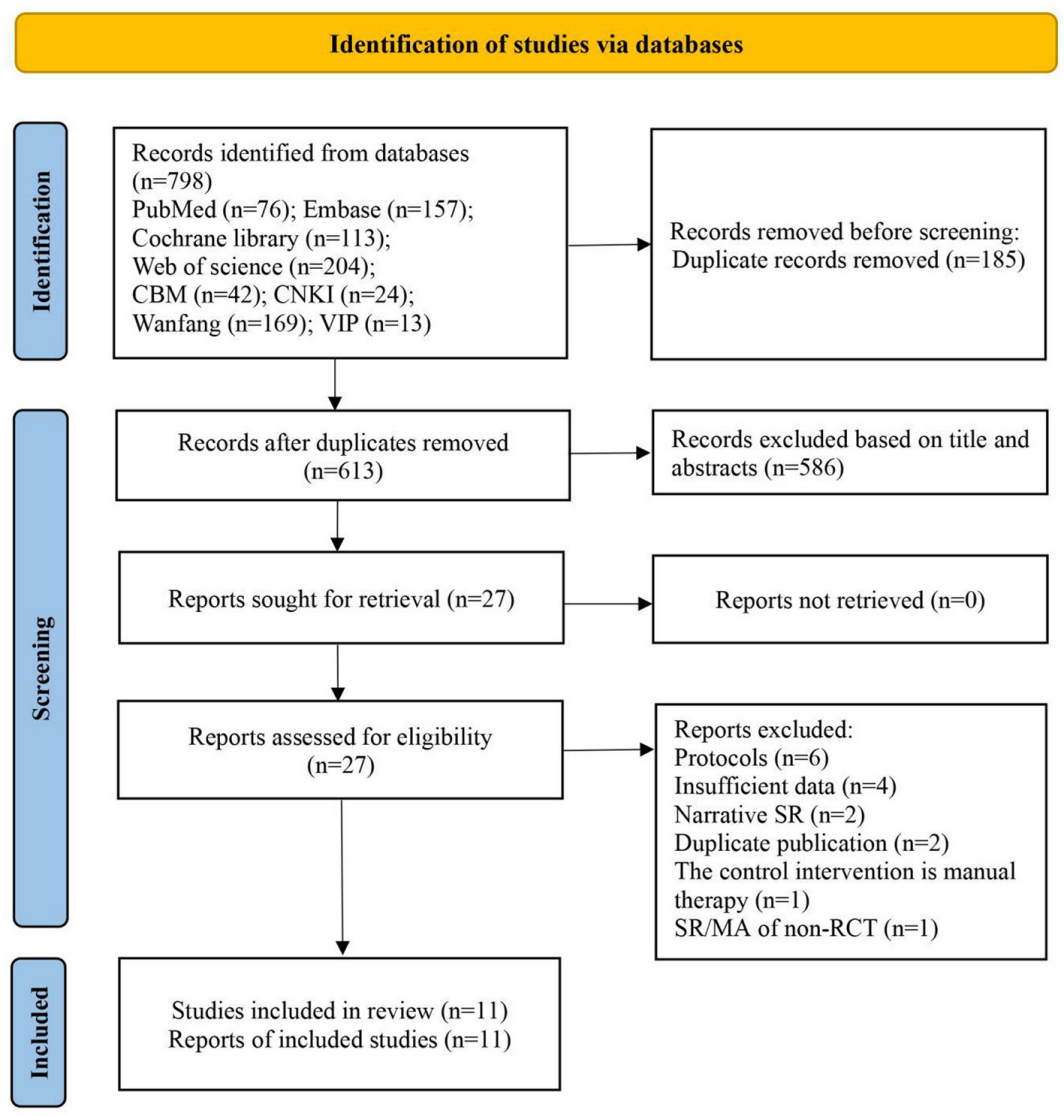
The PRISMA flow diagram of study selection.

A total of 798 records were accessed from eight electronic databases. After removing duplication, 185 records were excluded. After screening titles and abstracts, 586 records were excluded. Meanwhile, 27 SRs/MAs needed to be further screened by reviewing the full text. Finally, eleven SRs/MAs (48–58) were included in this overview (Figure 2). The reasons for exclusion were shown in Appendix F.

### 3.2. Characteristics of included SRs/MAs

Eleven reviews [four reviews (48, 49, 54, 56) in English and seven reviews (50–53, 55, 57, 58) in Chinese] were published from 2013 to 2022, including 5 to 29 RCTs whose number of subjects ranged from 451 to 2,920 individually. There are ten reviews (48–53, 55–58) from China and one (54) from Saudi Arabia. For the quality assessment tools, the Cochrane Collaboration Risk of Bias Tool was applied in five reviews (48, 49, 52, 53, 58), the PEDro scale was used in two reviews (54, 56), the Jadad scale was used in one review (50), and the Cochrane Collaboration Risk of Bias Tool combined with the Jadad scale were adopted in three reviews (51, 55, 57). As for intervention, all reviews compared manual therapy with controls, which included sham (placebo) manual therapy, western medicine, acupuncture therapy, exercise therapy, usual care, or conventional treatments. More details can be found in Table 2.

**Table 2:** Characteristics of the included SRs/Mas.

**Authors (ref) country**	**Language**	**Number of included RCTs (simple Size)**	**Intervention**	**Comparator**	**Quality assessment tools**	**Duration of treatment**	**Outcomes**	**Overall conclusions (quote from the original paper)**	**Adverse effects**
Wu et al. (48) China	English	12 (737)	MT	UC/SMT/CT/ Medication	Cochrane Collaboration Risk of Bias Tool	1–16 weeks	B; C; D; E	“Massage therapy may lead to some improvement in pain, stiffness, and functionality scores in the short term but not in long term”	Not reported
Xing et al. (49) China	English	8 (711)	MT	Medication	Cochrane Collaboration Risk of Bias Tool	2–4 weeks	A; B; C; D	“Therapeutic massage was more effective than oral NSAIDs in treating KOA. In relieving pain and stiffness and improving the function of knee joint, therapeutic massage was superior to NSAIDs”	Not reported
Zhang et al. (50) China	Chinese	8 (632)	MT	Medication/AT	Jadad scale	2–4 weeks	A	“The existing clinical evidence shows that massage alone can effectively alleviate the clinical symptoms of KOA patients and improve their quality of life”	Yes
Xu et al. (51) China	Chinese	17 (1,387)	MT	Medication/AT	Cochrane Collaboration Risk of Bias Tool; Jadad scale	2–6 weeks	A; E	“Compared with western medicine, both groups show equivalent effects on VAS score and adverse reactions, and Chinese Tuina was better than western medicine in improving WOMAC score. Chinese Tuina was better than the control group in improving the effective rate”	Yes
Tang et al. (52) China	Chinese	26 (2,920)	MT	AT/ET/CT/ Medication	Cochrane Collaboration Risk of Bias Tool	10 days-6 weeks	A; E; F	“The effect of massage on KOA was better, and the improvement of VAS score, WOMAC score, HSS score, etc. was not weaker than that of western medicine, traditional medicine, acupuncture or joint cavity injection”	Yes
Yu et al. (53) China	Chinese	6 (590)	MT	AT	Cochrane Collaboration Risk of Bias Tool	2–4 weeks	B; C; D	“In improving the stiffness of the joint, the traditional Chinese manipulation is superior to the acupuncture, and the effect of both and physical function is equivalent”	Not reported
Anwer et al. (54) Saudi Arabia	English	11 (494)	MT	ET	PEDro scale	2–24 weeks	B; D; E; G	“This review indicated orthopedic manual therapy compared with exercise therapy alone provides short-term benefifits in reducing pain, improving function, and physical performance in patients with KOA”	Not reported
Yu et al. (55) China	Chinese	29 (2,678)	MT	Medication/ AT/UC	Cochrane Collaboration Risk of Bias Tool; Jadad scale	10 days-8 weeks	A; B; C; D; E	“Chinese manipulation is effective in treating knee osteoarthritis, and the total effective rate is better than other conservative treatment. In the aspect of improving pain and stiffness, Chinese manipulation has advantages over acupuncture, Chinese manipulation is superior to western medicine in improving joint function”	Not reported
Xu et al. (56) China	English	14 (841)	MT	UC/AT/ET/ Medication	PEDro scale	2–12 weeks	B; C; D	“The preliminary evidence from our study suggests that manual therapy might be effective and safe for improving pain, stiffness, and physical function in KOA patients and could be treated as complementary and alternative options”	Yes
Cai et al. (57) China	Chinese	14 (1,174)	MT	Medication/ AT/CT	Cochrane Collaboration Risk of Bias Tool; Jadad scale	15 days-8 weeks	A; E	“The total effective rate of the evaluation criteria manipulation in the treatment of osteoarthritis of the knee significantly better than medicines and acupuncture treatment by Meta-analysis”	Not reported
Wang et al. (58) China	Chinese	5 (451)	MT	UC/ET/SMT	Cochrane Collaboration Risk of Bias Tool	4–48 weeks	E	“Manipulation therapy has therapeutic effect on alleviating knee joint pain and improving the knee joint activity both in short and long term”	Not reported

MT, manual therapy; AT, acupuncture therapy; ET, exercise therapy; UC, usual care; CT, conventional treatment; SMT, sham manual therapy; A, total effective rate; B, WOMAC pain score; C, WOMAC stiffness score; D, WOMAC function score; E, Visual Analog Scale (VAS); F, hospital for special surgery knee score (HSS); G, stairs ascending-descending time.

### 3.3. Methodological quality of included SRs/MAs

By AMSTAR-2, the methodological quality of nine reviews (48–53, 56–58) was assessed as critically low quality, and two (54, 55) were rated low. Their deficiencies were as follows: For item 2, ten reviews (48–53, 55–58) were not registered in advance, and the protocol wasn't published before conducting the study. For item 3, six reviews (49–51, 54, 56, 58) didn't explain the reasons for choosing RCTs to include the study. For item 4, only three reviews (48, 49, 54) described a comprehensive literature search strategy. Gray literature retrieval was ignored in some studies. For item 7, seven reviews (48, 50–53, 56, 57) didn't provide a list of excluded studies. For item 8, none of the reviews described the dose of intervention. For item 10, none of the reviews described the funding sources of the included RCTs. For item 12, ten reviews (48–54, 56–58) ignored the potential impact of bias on the meta-analysis result in each trial. For item 14, five reviews (50, 52, 53, 57, 58) didn't explain reasons for heterogeneity reasonably. For item 15, six reviews (48, 49, 52–54, 58) didn't adequately explore the publication bias. For item 16, eight reviews (50–53, 55–58) didn't declare any conflicts of interest or provide the source of funding. More details were shown in Table 3.

**Table 3:** Results of the AMSTAR-2 assessments.

**Items**	**Wu et al. (48)**	**Xing et al. (49)**	**Zhang et al. (50)**	**Xu et al. (51)**	**Tang et al. (52)**	**Yu et al. (53)**	**Anwer et al. (54)**	**Yu et al. (55)**	**Xu et al. (56)**	**Cai et al. (57)**	**Wang et al. (58)**
1	Y	Y	Y	Y	Y	Y	Y	Y	Y	Y	Y
2	N	N	N	N	N	N	Y	N	N	N	N
3	Y	N	N	N	Y	Y	N	Y	N	Y	N
4	Y	Y	PY	PY	PY	PY	Y	PY	PY	PY	PY
5	Y	Y	Y	Y	Y	Y	Y	Y	Y	Y	Y
6	Y	Y	Y	Y	Y	Y	Y	Y	Y	Y	Y
7	N	Y	N	N	N	N	Y	Y	N	N	Y
8	PY	PY	PY	PY	PY	PY	PY	PY	PY	PY	PY
9	Y	Y	Y	Y	Y	Y	Y	Y	Y	Y	Y
10	N	N	N	N	N	N	N	N	N	N	N
11	Y	Y	Y	Y	Y	Y	Y	Y	Y	Y	Y
12	N	N	N	N	N	N	N	Y	N	N	N
13	Y	Y	Y	Y	Y	Y	Y	Y	Y	Y	Y
14	Y	Y	N	Y	N	N	Y	Y	Y	N	N
15	N	N	Y	Y	N	N	N	Y	Y	Y	N
16	Y	Y	N	N	N	N	Y	N	N	N	N
Methodological quality	Critically low	Critically low	Critically low	Critically low	Critically low	Critically low	Low	Low	Critically low	Critically low	Critically low

Y, Yes; PY, Partial Yes; N, No.

### 3.4. Risk of bias of included SRs/MAs

According to the rules of ROBIS, all reviews were assessed as low risk in Phase 1 and Domain 1. For Domain 2, three reviews (48, 49, 54) (27.27%) were rated low risk, and eight reviews (50–53, 55–58) (72.73%) were assessed as high risk. For Domain 3, ten reviews (48, 49, 51–58) (90.91%) were rated low risk, and one (50) (9.10%) was assessed as high risk. For Domain 4, five reviews (48, 49, 54–56) (45.45%) were rated low risk, five reviews (50, 52, 53, 57, 58) (45.45%) were rated high risk, and one (51) (9.10%) was unclear risk. For Phase 3, five reviews (48, 49, 54–56) (45.45%) were rated low risk and six (50–53, 57, 58) (54.55%) were high risk. More details were shown in Table 4.

**Table 4:** Results of ROBIS assessments.

**Review**	**Phase 1**	**Phase 2**				**Phase 3**
	**Assessing relevance**	**Domain 1. Study eligibility criteria**	**Domain 2. Identification and selection of studies**	**Domain 3. Data collection and study appraisal**	**Domain 4. Synthesis and findings**	**Risk of bias in the review**
Wu et al. (48)	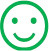	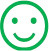	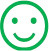	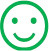	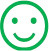	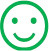
Xing et al. (49)	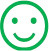	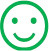	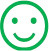	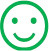	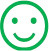	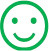
Zhang et al. (50)	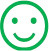	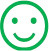	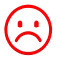	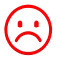	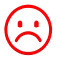	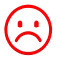
Xu et al. (51)	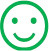	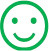	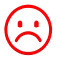	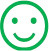	?	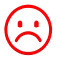
Tang et al. (52)	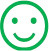	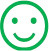	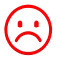	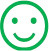	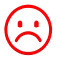	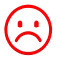
Yu et al. (53)	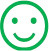	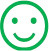	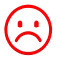	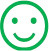	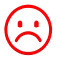	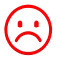
Anwer et al. (54)	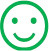	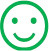	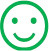	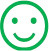	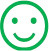	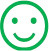
Yu et al. (55)	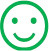	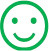	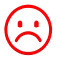	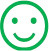	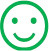	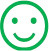
Xu et al. (56)	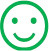	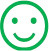	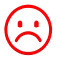	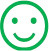	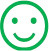	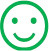
Cai et al. (57)	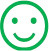	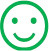	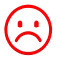	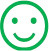	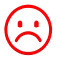	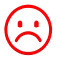
Wang et al. (58)	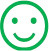	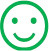	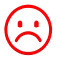	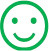	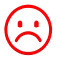	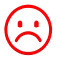

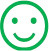
: Low risk; 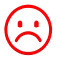: High risk; ? : Unclear.

### 3.5. Reporting quality of included SRs/MAs

**Figure 3:**
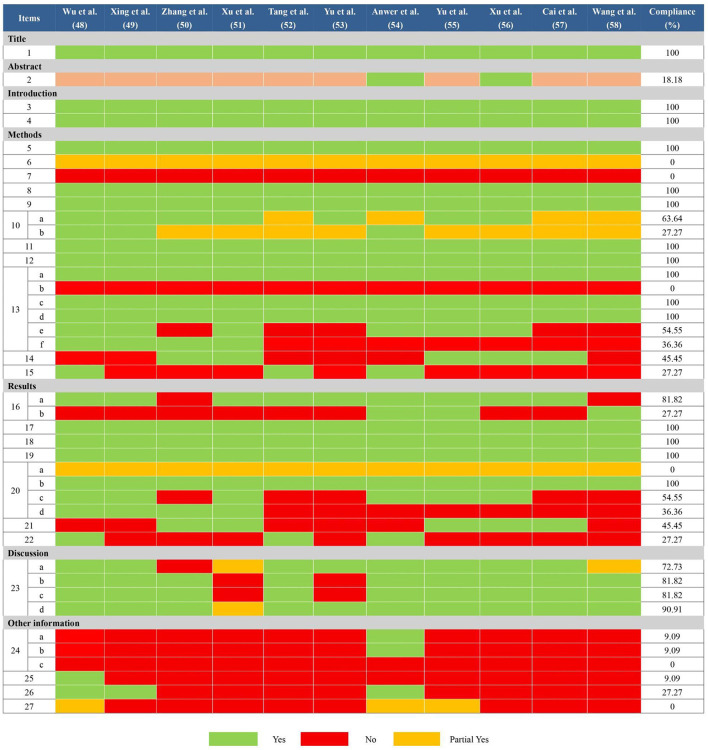
Results of the PRISMA 2020 assessments. Item 1: Title; Item 2: Abstract; Item 3: Rationale; Item 4: Objectives; Item 5: Eligibility criteria; Item 6: Information sources; Item 7: Search strategy; Item 8: Selection process; Item 9: Data collection process; Item 10: Data items; Item 11: Study risk of bias assessment; Item 12: Effect measures; Item 13: Synthesis methods; Item 14: Reporting bias assessment; Item 15: Certainty assessment; Item 16: Study selection; Item 17: Study characteristics; Item 18: Risk of bias in studies; Item 19: Results of individual studies; Item 20: Results of syntheses; Item 21: Reporting biases; Item 22: Certainty of evidence; Item 23: Discussion; Item 24: Registration and protocol; Item 25: Support; Item 26: Competing interests; Item 27: Availability of data, code, and other materials.

In general, there are some deficiencies in the reporting process. Many reviews (48–53, 55, 57, 58) didn't provide adequate information according to the abstract checklist (Q2: 18.18%). In terms of methods, all reviews didn't report comprehensively the information sources and search strategies in all databases (Q6: 0%; Q7: 0%). All reviews didn't mention preprocessing of merging data (Q13b: 0%). Partial reviews didn't report how to explore the heterogeneity (50, 52, 53, 57, 58) or analyze the sensitivity (52–58) (Q13e: 54.55%; Q13f: 36.36%). Partial reviews didn't explore the publication bias (48, 49, 52–54, 58) and evidence quality (49–51, 53, 55–58) (Q14: 45.45%; Q15: 27.27%). Besides, in the section on results, some reviews (48–53, 56, 57) didn't provide the list of excluded references and reasons (Q16b: 27.27%). All reviews didn't report the risk of bias in each meta-analysis result (Q20a: 0%) and partial reviews didn't report the results of heterogeneity sources (50, 52, 53, 57, 58) and sensitivity analyses (52–58) (Q20c: 54.55%; Q20d: 36.36%). Partial reviews didn't report the results of publication bias (48, 49, 52–54, 58) and evidence quality (49–51, 53, 55–58) (Q21: 45.45%; Q22: 27.27%). Furthermore, as for other information, only one review (54) had been registered before conducting their study (Q24a: 9.09%; Q24b: 9.09%) and none of the reviews mentioned a revision of their protocol (Q24c: 0%). Most of the reviews ignored the descriptions of funding (49–58) and conflict of interest (50–53, 55–58) (Q25: 9.09%; Q26: 27.27%). None of the reviews mentioned access to relevant information (Q27: 0%). More details were shown in Figure 3.

### 3.6. Evidence quality of included SRs/MAs

The evidence quality of 38 items in eleven SRs/MAs was defined as moderate quality (8 items, 21.05%), low quality (16 items, 42.11%), and critically low quality (14 items, 36.84%). Risk of bias (38 items, 100%), inconsistency (20 items, 52.63%), imprecision (18 items, 47.37%), and publication bias (8 items, 21.05%) were the main downgraded factors. More details were shown in Table 5.

**Table 5:** Results of the GRADE.

**Outcome measures**	**No. of RCTs (No. of participants)**	**Interventions**	**Effect estimate 95% CI**	***p*-value**	**Risk of bias**	**Inconsistency**	**Indirectness**	**Imprecision**	**Publication bias**	**Quality of evidence**	**References**
Total effective rate	7 (661)	MT vs. Medication	RR 1.14 (1.07, 1.21)	< 0.00001	−1	0	0	0	0	M	(49)
	8 (632)	MT vs. Medication/AT	OR 2.03 (1.43, 2.88)	< 0.00001	−1	0	0	0	0	M	(50)
	16 (1,307)	MT vs. Medication/AT	OR 2.30 (1.65, 3.22)	< 0.00001	−1	0	0	0	0	M	(51)
	7 (728)	MT vs. Medication	RR 1.13 (1.06, 1.20)	< 0.00001	−1	0	0	0	0	M	(52)
	2 (229)	MT vs. ET	RR 1.34 (1.13, 1.59)	0.0009	−1	0	0	−1	−1	CL	(52)
	23 (2,144)	MT vs. AT/UC/Medication	OR 3.26 (2.48, 4.29)	< 0.00001	−1	0	0	0	0	M	(55)
	12 (909)	MT vs. AT/CT/Medication	OR 2.46 (1.57, 3.86)	< 0.00001	−1	0	0	0	0	M	(57)
VAS	4 (167)	MT vs. Medication/UC/SMT	SMD −0.09 (−0.49, 0.30)	0.64	−1	0	0	−1	0	L	(48)
	5 (361)	MT vs. Medication	SMD −0.33 (−0.93, 0.28)	0.29	−1	−1	0	−1	0	CL	(51)
	3 (288)	MT vs. Medication	SMD −0.38 (−1.52, 0.75)	0.51	−1	−1	0	−1	−1	CL	(52)
	4 (300)	MT vs. UC/CT/LT	SMD −1.95 (−3.78, −0.12)	0.04	−1	−1	0	−1	0	CL	(48)
	5 (141)	MT vs. ET	SMD −0.80 (−1.43, −0.17)	0.013	−1	−1	0	−1	0	CL	(54)
	15 (1,434)	MT vs. AT/Medication/UC	SMD 0.98 (0.23, 1.74)	0.01	−1	−1	0	0	0	L	(55)
	6 (437)	MT vs. AT/CT/Medication	SMD 0.47 (0.30, 0.64)	< 0.00001	−1	0	0	0	0	M	(57)
	2 (244)	MT vs. UC/SMT	SMD −8.29 (−15.70, −0.88)	0.03	−1	0	0	−1	0	L	(58)
	4 (283)	MT vs. UC/SMT	SMD −16.64 (−22.15, −11.12)	< 0.00001	−1	0	0	−1	0	L	(58)
WOMAC pain score	6 (590)	MT vs. AT	SMD 0.79 (0.01, 1.57)	0.05	−1	−1	0	0	0	L	(53)
	6 (426)	MT vs. UC/SMT	SMD −1.24 (−2.22, 0.28)	0.11	−1	−1	0	0	0	L	(48)
	3 (132)	MT vs. ET	SMD −0.79 (−1.14, −0.44)	0.001	−1	0	0	−1	−1	CL	(54)
	14 (1,192)	MT vs. AT/Medication/UC	SMD 0.68 (0.23, 1.13)	0.003	−1	−1	0	0	0	L	(55)
	11 (657)	MT vs. Medication/UC/AT	SMD −0.61 (−0.95, −0.28)	0.0003	−1	−1	0	0	0	L	(56)
	5 (227)	MT vs. UC/CT/SMT	SMD −1.96 (−3.25, −0.68)	0.003	−1	−1	0	−1	0	CL	(48)
	2 (135)	MT vs. Medication	SMD −2.06 (−2.75, −1.36)	< 0.00001	−1	0	0	−1	−1	CL	(49)
WOMAC stiffness score	4 (345)	MT vs. Medication	SMD −0.90 (−1.05, −0.75)	< 0.00001	−1	0	0	−1	0	L	(49)
	6 (590)	MT vs. AT	SMD 0.66 (0.06, 1.27)	0.03	−1	−1	0	0	0	L	(53)
	13 (1,132)	MT vs. AT/Medication/UC	SMD 0.50 (0.13, 0.87)	0.008	−1	−1	0	0	0	L	(55)
	11 (657)	MT vs. Medication/UC/AT	SMD −0.58 (−0.95, −0.21)	0.002	−1	−1	0	0	0	L	(56)
	5 (227)	MT vs. UC/CT/SMT	SMD −0.60 (−1.00, −0.20)	0.003	−1	−1	0	−1	0	CL	(48)
	6 (426)	MT vs. UC/SMT	SMD −0.80 (−1.45, −0.16)	0.01	−1	−1	0	0	0	L	(48)
WOMAC function score	4 (460)	MT vs. AT	SMD 0.59 (−0.09, 1.26)	0.09	−1	−1	0	0	0	L	(53)
	10 (882)	MT vs. AT/Medication/UC	SMD 0.75 (0.32, 1.18)	0.0007	−1	−1	0	0	0	L	(55)
	11 (657)	MT vs. Medication/UC/AT	SMD −0.49 (−0.76, −0.22)	0.0004	−1	−1	0	0	0	L	(56)
	3 (132)	MT vs. ET	SMD −0.85 (−1.20, −0.50)	0.001	−1	0	0	−1	−1	CL	(54)
	3 (225)	MT vs. Medication	SMD −12.48 (−13.91, −11.05)	< 0.00001	−1	0	0	−1	−1	CL	(49)
	6 (426)	MT vs. UC/SMT	SMD −1.50 (−2.14, −0.87)	< 0.00001	−1	0	0	0	0	M	(48)
	6 (287)	MT vs. UC/CT/LT	SMD −2.57 (−5.39, 0.25)	0.07	−1	−1	0	−1	0	CL	(48)
HSS	2 (210)	MT vs. Medication	SMD 6.41 (−5.92, 18.74)	0.31	−1	−1	0	−1	−1	CL	(52)
Stairs ascending-descending time	2 (48)	MT vs. ET	SMD −0.88 (−1.48, −0.29)	0.004	−1	0	0	−1	−1	CL	(54)

MT, manual therapy; AT, acupuncture therapy; ET, exercise therapy; UC, usual care; CT, conventional treatment; SMT, Sham manual therapy; RR, risk ratio; OR, odds ratio; SMD, standard mean difference; 95% CI, 95% confidence interval; The design of the experiment with a large bias in random, distributive hiding or blind; The confidence interval overlaps less, the heterogeneity test P is critically small, and the I^2^ is larger; Confidence interval is not narrow enough; Fewer studies are included and there may be greater publication bias; M, moderate quality; L, Low quality; CL, Critically low quality.

### 3.7. Outcomes and efficacy evaluation

#### 3.7.1. Total effective rate

**Figure 4:**
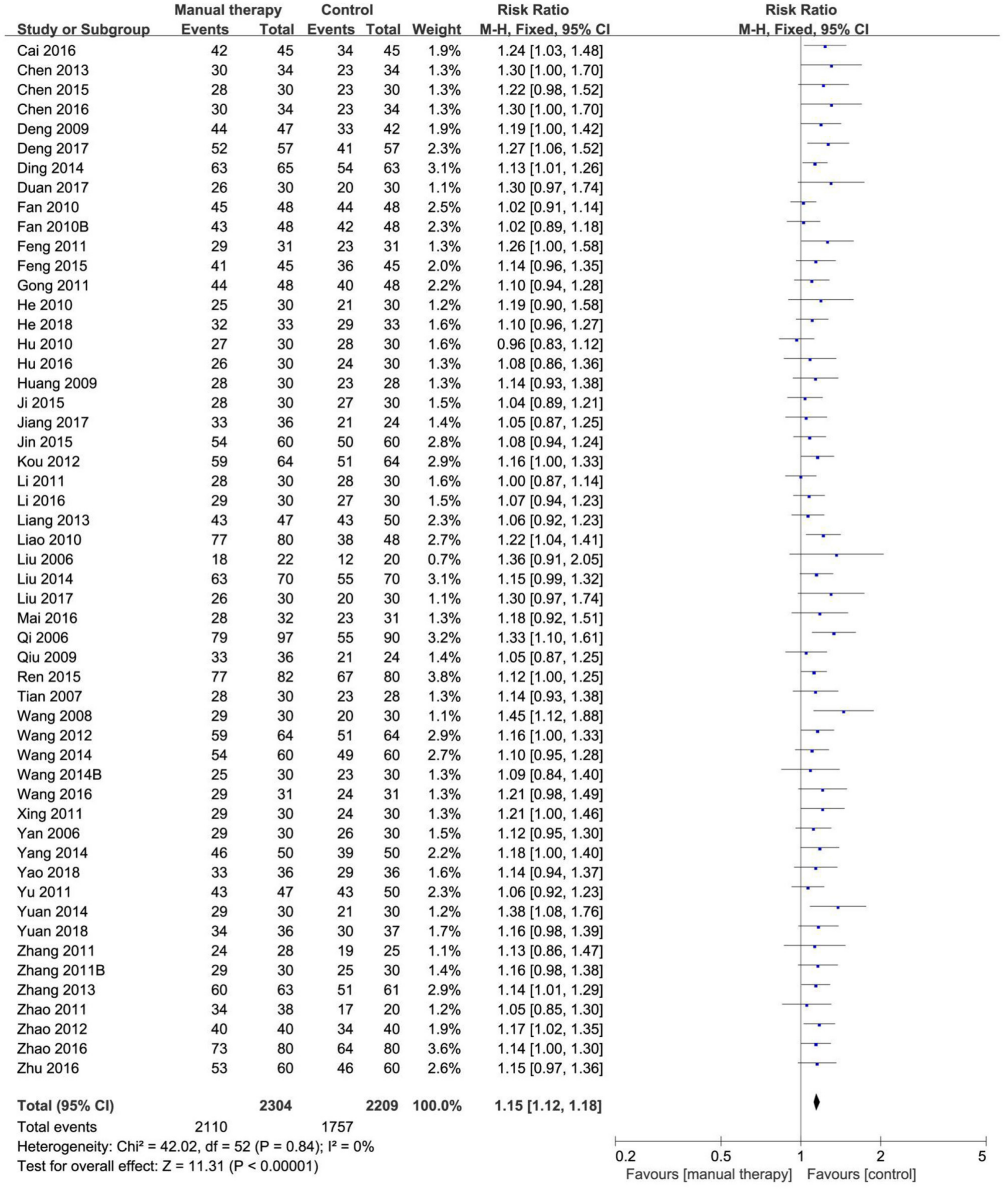
Forest plot of total effective rate.

**Figure 5:**
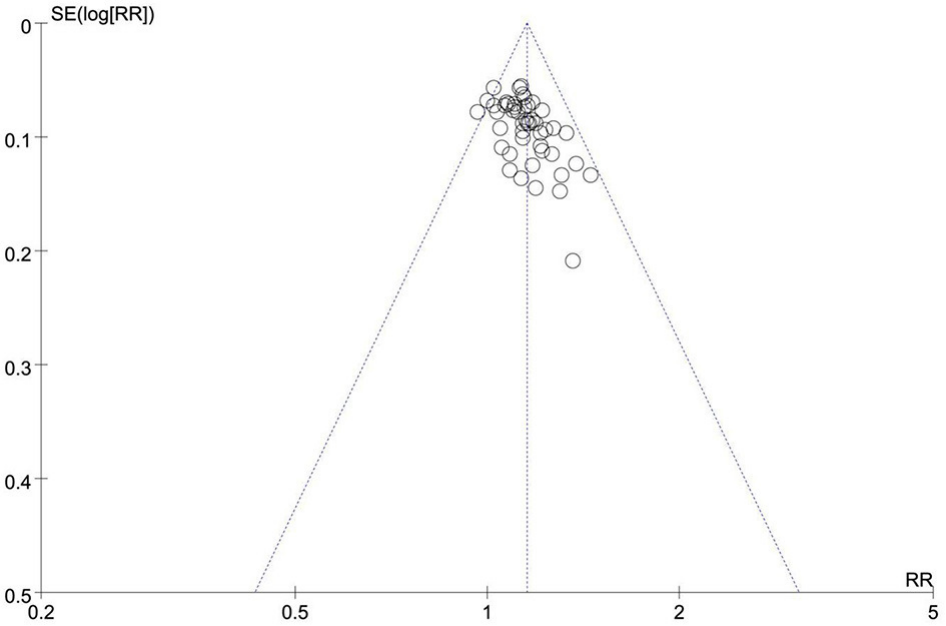
Funnel plot of total effective rate.

Six reviews (49–52, 55, 57) compared the effects of manual therapy with those of medication, acupuncture therapy, exercise therapy, usual care, or conventional treatment using the total effective rate. In all six SRs/MAs, manual therapy appeared to be more effective than control interventions in terms of the total effective rate. One review (52) reported a higher total effective rate with manual therapy than medication (RR = 1.13, 95% CI [1.06, 1.20], *p* < 0.00001) and exercise therapy (RR = 1.34, 95% CI [1.13, 1.59], *p* = 0.0009). Two reviews (50, 51) found that, as compared with medication or acupuncture therapy, manual therapy was associated with a higher total effective rate (OR = 2.03, 95% CI [1.43, 2.88], *p* < 0.00001; OR = 2.30, 95% CI [1.65, 3.22], *p* < 0.00001). Three reviews (49, 55, 57) showed a significant effect of manual therapy compared with medication, acupuncture therapy, usual care, or conventional treatment (RR = 1.14, 95% CI [1.07, 1.21], *p* < 0.00001; OR = 3.26, 95% CI [2.48, 4.29], *p* < 0.00001; OR = 2.46, 95% CI [1.57, 3.86], *p* < 0.00001). We conducted a re-meta-analysis on the total effective rate. A total of 53 RCTs (4,513 participants) were included after the overlapping RCTs were removed. The result showed no homogeneity among these studies (I^2^= 0, *p* = 0.84). The effect of manual therapy for KOA was better than that of control interventions on the total effective rate (RR = 1.15, 95% CI [1.12, 1.18], *p* < 0.00001; Figure 4). The funnel plot showed no obvious publication bias (Figure 5).

#### 3.7.2. VAS score

Five reviews (48, 54, 55, 57, 58) reported lower scores for manual therapy on the VAS. One review (58) showed a favorable effect of manual therapy compared with usual care or sham manual therapy (long term: SMD = −8.92, 95% CI [−15.70, −0.88], *p* = 0.03; short term: SMD = −16.64, 95% CI [−22.15, −11.12], *p* < 0.00001). Another four reviews (48, 54, 55, 57) also found a significant effect of manual therapy compared to control interventions (SMD = −1.95, 95% CI [−3.78, −0.12], *p* = 0.04; SMD = −0.80, 95% CI [−1.43, −0.17], *p* = 0.013; SMD = 0.98, 95% CI [0.23, 1.74], *p* = 0.01; SMD = 0.47, 95% CI [0.30, 0.64], *p* < 0.00001). Whereas, Wu et al. (48) indicated no significant reduction of VAS with 4 weeks compared to control groups (SMD = −0.09, 95% CI [−0.49, 0.30], *p* = 0.64). Xu et al. (51) and Tang et al. (52) showed no significant effect on the VAS of manual therapy compared to medication (SMD = −0.33, 95% CI [−0.93, 0.28], *p* = 0.29; SMD = −0.38, 95% CI [−1.52, 0.75], *p* = 0.51).

#### 3.7.3. WOMAC pain score

Five reviews (48, 49, 54–56) reported significant effect on the WOMAC pain score of manual therapy compared to control groups (SMD = −0.79, 95% CI [−1.14, −0.44], *p* = 0.001; SMD = 0.68, 95% CI [0.23, 1.13], *p* = 0.003; SMD = −0.61, 95% CI [−0.95, −0.28], *p* = 0.0003; SMD = −1.96, 95% CI [−3.25, −0.68], *p* = 0.003; SMD = −2.06, 95% CI [−2.75, −1.36], *p* < 0.00001). However, Wu et al. (48) indicated no significant difference in WOMAC pain scores between the two groups after six to eight weeks (SMD = −1.24, 95% CI [−2.22, 0.28], *p* = 0.11). Yu et al. (53) found no significant difference on the WOMAC pain score between manual therapy and acupuncture therapy (SMD = 0.79, 95% CI [0.01, 1.57], *p* = 0.05).

#### 3.7.4. WOMAC stiffness score

Five reviews (48, 49, 53, 55, 56) found a significant difference in WOMAC stiffness score compared to control groups. One review (48) revealed a statistically significant reduction in stiffness scores with 4 weeks (SMD = −0.60, 95% CI [−1.00, −0.20], *p* = 0.003) or 6–8 weeks of manual therapy (SMD = −0.80, 95% CI [−1.45, −0.16], *p* = 0.01). Besides, Another four reviews (49, 53, 55, 56) also found significant difference in improving stiffness scores between manual therapy and control groups (SMD = −0.90, 95% CI [−1.05, −0.75], *p* < 0.00001; SMD = 0.66, 95% CI [0.06, 1.27], *p* = 0.03; SMD = 0.50, 95% CI [0.13, 0.87], *p* = 0.008; SMD = −0.58, 95% CI [−0.95, −0.21], *p* = 0.002).

#### 3.7.5. WOMAC function score

Five reviews (48, 49, 54–56) reported a favorable effect of manual therapy on WOMAC function score compared to control groups (SMD = 0.75, 95% CI [0.32, 1.18], *p* = 0.0007; SMD = −0.49, 95% CI [−0.76, −0.22], *p* = 0.0004; SMD = −0.85, 95% CI [−1.20, −0.50], *p* = 0.001; SMD = −12.48, 95% CI [−13.91, −11.05], *p* < 0.00001; SMD = −1.50, 95% CI [−2.14, −0.87], *p* < 0.00001). Nevertheless, Wu et al. (48) found no significant difference between the two groups after 4 weeks (SMD = −2.57, 95% CI [−5.39, 0.25], *p* = 0.07). Yu et al. (53) revealed no statistical difference in improving function score compared to acupuncture therapy (SMD = 0.59, 95% CI [−0.09, 1.26], *p* = 0.09).

#### 3.7.6. HSS and stairs ascending-descending time

Tang et al. (52) found no significant difference on the HSS between manual therapy and medication (SMD = 6.41, 95% CI [−5.92, 18.74], *p* = 0.31). Anwer et al. (54) showed a significant effect on stairs ascending-descending time compared to exercise therapy (SMD = −0.88, 95% CI [−1.48, −0.29], *p* = 0.004) based on the insufficient number of subjects (total 48).

#### 3.7.7. Adverse effects

Although none of the included reviews provides a quantitative comparison of adverse effects between manual therapy and the control group, four SRs/MAs (50–52, 56) narratively reported and discussed this aspect. The findings regarding adverse effects indicated that there were no severe adverse reactions in the manual therapy group.

## 4. Discussion

### 4.1. Research significance of this overview

With the increased awareness of the adverse effects of drugs, there has been a heightened interest in complementary and alternative treatments for KOA (59, 60). Manual therapy has gradually derived a variety of applications for patients with KOA in recent years (61–65). Numerous trils (66–69) and SRs/MAs (48–58) have been performed to investigate the effectiveness and safety of manual therapy. However, a few SRs/MAs were reported in accordance with PRISMA 2020 or assessed for quality of evidence by GRADE. The methodological quality and risk of bias of SRs/MAs were still uncertain. Therefore, it is inadequate to guide clinical practice based on individual SR/MA or low quality SRs/MAs with unconvincing conclusions. Under the circumstances, the establishment of a comprehensive overview of these SRs/MAs can more effectively guide clinical practice. Besides, the deficiencies and gaps in the overview may provide notable information and direction for future studies. Thus, it is significant and innovative to conduct a systematic overview based on these SRs/MAs.

### 4.2. Pivotal findings of this overview

Firstly, we found that manual therapy was significantly superior to the control group in terms of the total effective rate. But in terms of improving pain and function in KOA patients, the included reviews draw inconsistent conclusions, possibly due to different control interventions, treatment durations, disease severity, and the number of subjects. Some reviews conducted quantitative synthesis on different control measures or durations of manual therapy, but most of the included reviews did not differentiate specifically between these factors or lacked subgroup analysis. As for the safety of manual therapy, the results indicated that there were no severe adverse reactions in the manual therapy group, which revealed that manual therapy may be a safe complementary and alternative treatment. Nevertheless, the promotion and application of these results are limited by the overall quality of the included reviews. The methodological quality of included reviews were rated critically low or low quality. Partial reviews were assessed as high risk in Phase 2 and Phase 3 by ROBIS. Therefore, since definitive conclusions can't be drawn in accordance with published results, caution is warranted when recommending manual therapy as an alternative treatment for improving the symptoms of KOA patients. SRs/MAs with high methodological quality and low risk of bias are needed to evaluate the effectiveness and safety of manual therapy.

Secondly, there is much room for addressing quality during the SR/MA process. In the case of AMSTAR-2, only one review registered a protocol of preliminary design, which may affect the transparency of the study and increase the risk of bias. All the authors conducted literature searches in multiple databases, whereas most of them didn't apply a complete search strategy and provide a list of excluded studies. Partial reviews didn't reasonably explore the heterogeneity and publication bias. And some reviews didn't declare the source of funding. According to ROBIS, a few reviews were assessed as low risk, particularly in Domain 2 of Phase 2 and Phase 3. From the PRISMA 2020 results, the included reviews had different reporting flaws, including mainly these aspects: abstract, protocol and registration, preprocessing of merging data, heterogeneity analysis, sensitivity analysis, publication bias, evidence quality, the list of excluded references, funding source, conflict of interest, and the approach to relevant information. The poor reporting quality may exaggerate the effectiveness of manual therapy, which may diminish the value of the design. Based on GRADE, the evidence of low and critically low quality accounted for 78.95%. The risk of bias in the RCTs was the most common factor degrading the level of evidence. A large proportion of the RCTs had an unclear risk of bias for random sequence generation, allocation concealment, and blinding. Inconsistency may exist due to a large number of clinical characteristics and methodological differences in the RCTs, which could result in high heterogeneity. Moreover, the implausible study designs and small sample size may cause imprecision and publication bias. These deficiencies will provide a promising direction for future researchers to promote the generation of advanced evidence.

### 4.3. Implications for further studies

This overview introduces several challenges for producers of RCTs and SRs/MAs that should be taken into consideration: (a) Manual therapy appears to be superior to the control group in improving the total effective rate, but the effect on improving pain and physical function is still needed to be further explored. In addition, more normative SRs/MAs are required to evaluate the short and long term effects of manual therapy based on different durations. Researchers should pay attention to the dose-effect and time-effect relationships of manual therapy for KOA. (b) Reviewers should register or publish the research protocol of the preliminary design at PROSPERO, Cochrane, or a public publication in advance for a transparent process. (c) A comprehensive search strategy and a list of excluded studies with explanations should be provided. The gray literature should be taken into account when reviewers conduct searches. (d) The conflicts of interest and funding source should be mentioned in the article. (e) If the heterogeneity is significant, subgroup analysis or meta-regression should be conducted. Reviewers shouldn't also ignore the exploration of publication bias, sensitivity analysis, and evidence quality. (f) Most of the RCTs included in the SRs/MAs had an unclear risk of bias for blinding, allocation concealment, and random sequence generation. So reviewers should comply with the relevant guidelines in order to minimize the bias. (g) We have insufficient evidence on the adverse effects of manual therapy, and researchers should further investigate its safety.

### 4.4. Strengths and limitations

To our knowledge, this is the first overview of SRs/MAs that focuses on the effectiveness and safety of manual therapy for KOA. AMSTAR-2, ROBIS, PRISMA 2020, and GRADE tools were used to comprehensively appraise the published SRs/MAs in a rigorous way, which was in favor of the clinical application. The revelent results may provide valuable evidence references for clinical practice, and promote the generation of advanced evidence of manual for KOA. However, several limitations in this overview were analyzed in the following: Firstly, we did not explore the influence of detailed control interventions and manual therapy durations because of deficient reporting in many SRs/MAs. This overview can not fully present situations in long term effect of manual therapy. Secondly, most of the included studies were conducted in China, so more studies should be performed to investigate whether the relevant conclusions can be generalized to other populations.

## 5. Conclusion

Manual therapy may be clinically effective and safe as a nonpharmacological intervention for KOA. Nevertheless, because of the generally unsatisfactory study quality and inconsistent conclusions of the SRs/MAs, this conclusion must be interpreted with caution. Further rigorous and normative SRs/MAs are expected to be carried out to provide robust evidence for definitive conclusions.

## Data availability statement

The original contributions presented in the study are included in the article/Supplementary material, further inquiries can be directed to the corresponding authors.

## Author contributions

Study conception and design: TF, LZ, and XWe. Final approval of manuscript materials: TF, LZ, and XWe. Collection and assembly of data: TF, XWa, ZJ, and XQ. Data analysis and interpretation: TF, CS, BQ, and YZ. Manuscript writing: TF and XWa. All authors contributed to the article and approved the submitted version.
