# Supplementary material for: Effectiveness and safety of manual therapy for knee osteoarthritis: An overview of systematic reviews and meta-analyses
Source: Front Public Health. 2023 Feb 24;11:1081238. doi: 10.3389/fpubh.2023.1081238 (PMC9999021; doi:10.3389/fpubh.2023.1081238)
Supplement: Supplementary file 1 [file Data_Sheet_1.pdf]

Supplementary Material

1 Appendix A: Search Strategies of Eight Databases

1.1 PubMed

#1 “Massage”[Mesh]

#2 “Manipulation, Osteopathic”[Mesh]

#3 “Chiropractic”[Mesh]

#4 “Osteoarthritis, Knee”[Mesh]

#5 “osteoarthritis”[Mesh]

#6 “Systematic reviews as topic”[Mesh]

#7 “Meta-analysis as topic”[Mesh]

#8 “Systematic review”[Publication Type]

#9 “Meta-analysis”[Publication Type]

#10 ((((((((((((((Zone Therapy[Title/Abstract]) OR (Zone Therapies[Title/Abstract])) OR (Massage Therapy[Title/Abstract])) OR (Massage Therapies[Title/Abstract])) OR (Osteopathic Manipulative Treatment[Title/Abstract])) OR (Osteopathic Manipulative Treatments[Title/Abstract])) OR (Osteopathic Manipulation[Title/Abstract])) OR (Tuina[Title/Abstract])) OR (manual therapy[Title/Abstract])) OR (manual traction[Title/Abstract])) OR (manipulation therapy[Title/Abstract])) OR (manipulative therapy[Title/Abstract])) OR (Massage[Title/Abstract])) OR (Manipulation, Osteopathic[Title/Abstract])) OR (Chiropractic[Title/Abstract])

#11 ((((((((((((Knee Osteoarthritides[Title/Abstract]) OR (Knee Osteoarthritis[Title/Abstract])) OR (Osteoarthritis of Knee[Title/Abstract])) OR (Osteoarthritis[Title/Abstract])) OR (Osteoarthritides[Title/Abstract])) OR (Osteoarthrosis[Title/Abstract])) OR (Osteoarthroses[Title/Abstract])) OR (Arthritis, Degenerative[Title/Abstract])) OR (Arthritides, Degenerative[Title/Abstract])) OR (Degenerative Arthritides[Title/Abstract])) OR (Degenerative Arthritis[Title/Abstract])) OR (Knee pain[Title/Abstract])) OR (KOA[Title/Abstract])

#12 (((((((((Meta-analysis[Title/Abstract]) OR (Systematic reviews[Title/Abstract])) OR (Systematic review[Title/Abstract])) OR (Meta analysis[Title/Abstract])) OR (Meta analyses[Title/Abstract])) OR (Meta-analyses[Title/Abstract])) OR (Evaluation of system[Title/Abstract])) OR (System assessment[Title/Abstract])) OR (System evaluation[Title/Abstract])) OR (Systematic assessment[Title/Abstract])

#13 #1 OR #2 OR #3 OR #10

#14 #4 OR #5 OR #11

#15 #6 OR #7 OR #8 OR #9 OR #12

#16 #13 AND #14 AND #15

1.2 EMbase

#1 'massage'/exp

#2 'osteopathic manipulation'/exp

#3 'chiropractic'/exp

#4 'knee osteoarthritis'/exp

#5 'osteoarthritis'/exp

#6 'systematic review'/exp

#7 'meta analysis'/exp

#8 'systematic review (topic)'/exp

#9 'meta analysis (topic)'/exp

#10 'chiropractic':ab,ti OR 'manipulation, osteopathic':ab,ti OR 'massage':ab,ti OR 'manipulative therapy':ab,ti OR 'manipulation therapy' OR 'manual traction':ab,ti OR 'manual therapy':ab,ti OR 'tuina':ab,ti OR 'osteopathic manipulation':ab,ti OR 'osteopathic manipulative treatments':ab,ti OR 'osteopathic manipulative treatment':ab,ti OR 'massage therapies':ab,ti OR 'massage therapy':ab,ti OR 'zone therapies':ab,ti OR 'zone therapy':ab,ti

#11 'degenerative arthritis':ab,ti OR 'degenerative arthritides':ab,ti OR 'arthritides, degenerative':ab,ti OR 'arthritis, degenerative':ab,ti OR 'osteoarthroses':ab,ti OR 'osteoarthrosis':ab,ti OR 'osteoarthritides':ab,ti OR 'osteoarthritis':ab,ti OR 'knee joint proliferative arthritis':ab,ti OR 'osteoarthritis of the knee':ab,ti OR 'osteoarthritis of knee':ab,ti OR 'knee osteoarthritis':ab,ti OR 'knee osteoarthritides':ab,ti OR 'knee pain':ab,ti OR 'KOA':ab,ti

#12 'systematic review':ab,ti OR 'systematic reviews':ab,ti OR 'meta analyses':ab,ti OR 'meta-analyses':ab,ti OR 'evaluation of system':ab,ti OR 'system assessment':ab,ti OR 'system evaluation':ab,ti OR 'systematic assessment':ab,ti

#13 #1 OR #2 OR #3 OR #10

#14 #4 OR #5 OR #11

#15 #6 OR #7 OR #8 OR #9 OR #12

#16 #13 AND #14 AND #15

1.3 Cochrane Library

#1 MeSH descriptor: [Massage] explode all trees

#2 MeSH descriptor: [Manipulation, Osteopathic] explode all trees

#3 MeSH descriptor: [Chiropractic] explode all trees

#4 MeSH descriptor: [Osteoarthritis, Knee] explode all trees

#5 MeSH descriptor: [Osteoarthritis] explode all trees

#6 MeSH descriptor: [Systematic reviews as topic] explode all trees

#7 MeSH descriptor: [Meta-analysis as topic] explode all trees

#8 (Zone Therapy):ti,ab,kw OR (Zone Therapies):ti,ab,kw OR (Massage Therapy):ti,ab,kw OR (Massage Therapies):ti,ab,kw OR (Osteopathic Manipulative Treatment):ti,ab,kw OR (Osteopathic Manipulative Treatments):ti,ab,kw OR (Osteopathic Manipulation):ti,ab,kw OR (Tuina):ti,ab,kw OR (manual therapy):ti,ab,kw OR (manual traction):ti,ab,kw OR (manipulation therapy):ti,ab,kw OR (manipulative therapy):ti,ab,kw OR (Massage):ti,ab,kw OR (Manipulation, Osteopathic):ti,ab,kw OR (Chiropractic):ti,ab,kw

#9 (Knee Osteoarthritides):ti,ab,kw OR (Knee Osteoarthritis):ti,ab,kw OR (Osteoarthritis of Knee):ti,ab,kw OR (Osteoarthritis of the Knee):ti,ab,kw OR (knee joint proliferative arthritis):ti,ab,kw OR (Osteoarthritis):ti,ab,kw OR (Osteoarthritides):ti,ab,kw OR (Osteoarthrosis):ti,ab,kw OR (Osteoarthroses):ti,ab,kw OR (Arthritis, Degenerative):ti,ab,kw OR (Arthritides, Degenerative):ti,ab,kw OR (Degenerative Arthritides):ti,ab,kw OR (Degenerative Arthritis):ti,ab,kw OR (Knee pain):ti,ab,kw OR (KOA):ti,ab,kw

#10 (Systematic review):ti,ab,kw OR (Meta-analysis):ti,ab,kw OR (Systematic reviews):ti,ab,kw OR (Meta analysis):ti,ab,kw OR (Meta analyses):ti,ab,kw OR (Meta-analyses):ti,ab,kw OR (Evaluation of system):ti,ab,kw OR (System assessment):ti,ab,kw OR (System evaluation):ti,ab,kw OR (Systematic assessment):ti,ab,kw

#11 #1 OR #2 OR #3 OR #8

#12 #4 OR #5 OR #9

#13 #6 OR #7 OR #10

#14 #11 AND #12 AND #13

1.4 Web of Science

#1 TS=(Massage OR Zone Therapy OR Therapies, Zone OR Zone Therapies OR Therapy, Zone OR Massage Therapy OR Therapies, Massage OR Massage Therapies OR Manipulation, Osteopathic OR Osteopathic Manipulative Treatment OR Osteopathic Manipulative Treatments OR Osteopathic Manipulation OR Tuina OR Chiropractic OR manual therapy OR manual

traction OR manual traction OR manipulation therapy OR manipulative therapy)

#2 TS=(Osteoarthritis, Knee OR Knee Osteoarthritides OR Knee Osteoarthritis OR Osteoarthritis of Knee OR Osteoarthritis of the Knee OR knee joint proliferative arthritis OR knee osteoarthritides OR Osteoarthritis OR Osteoarthrosis OR Osteoarthritides OR Osteoarthroses OR Degenerative Arthritides OR Degenerative Arthritis OR KOA OR knee pain)

#3 TS=(Systematic reviews as topic OR Meta-analysis as topic OR Systematic review OR Meta-analysis OR Meta analysis OR Meta analyses OR Meta-analyses OR Evaluation of system OR System assessment OR System evaluation OR Systematic assessment)

#4 #1 AND #2 AND #3

1.5 Chinese National Knowledge Infrastructure

(SU='手法' OR SU='推拿' OR SU='整脊' OR SU='正骨' OR SU='按摩') AND (SU='膝骨关节炎' OR SU='膝关节骨性关节炎' OR SU='膝骨性关节炎' OR SU='骨关节炎') AND (SU='系统评价' OR SU='meta 分析' OR SU='系统综述' OR SU='荟萃分析')

1.6 WanFang Database

(主题:(手法) or 主题:(推拿) or 主题:(正骨) or 主题:(整脊) or 主题:(按摩)) and (主题:(膝骨关节炎) or 主题:(膝关节骨性关节炎) or 主题:(膝骨性关节炎) or 主题:(骨关节炎)) and (主题:(系统评价) or 主题:(meta 分析) or 主题:(系统综述) or 主题:(荟萃分析))

1.7 Chongqing VIP Database

M=(手法 OR 推拿 OR 整脊 OR 正骨 OR 按摩) AND M=(膝骨关节炎 OR 膝关节骨性关节炎 OR 膝骨性关节炎 OR 骨关节炎) AND M=(系统评价 OR meta 分析 OR 系统综述 OR 荟萃分析)

1.8 Chinese Biological Medicine

#1 ("手法"[常用字段:智能] OR "推拿"[常用字段:智能] OR "整脊"[常用字段:智能] OR "正骨"[常用字段:智能] OR "按摩"[常用字段:智能])

#2 ("膝骨关节炎"[常用字段:智能] OR "膝关节骨性关节炎"[常用字段:智能] OR "膝骨性关节炎"[常用字段:智能] OR "骨关节炎"[常用字段:智能])

#3 ("系统评价"[常用字段:智能] OR "meta 分析"[常用字段:智能] OR "系统综述"[常用字段:智能] OR "荟萃分析"[常用字段:智能])

#4 #1 AND #2 AND #3

2 Appendix B: A Measurement Tool to Assess Systematic Reviews 2 (AMSTAR-2)

Item1: Did the research questions and inclusion criteria for the review include the components of PICO?

Item2: Did the report of the review contain an explicit statement that the review methods were established prior to the conduct of the review and did the report justify any signifificant deviations from the protocol?

Item3: Did the review authors explain their selection of the study designs for inclusion in the review?

Item4: Did the review authors use a comprehensive literature search strategy?

Item5: Did the review authors perform study selection in duplicate?

Item6: Did the review authors perform data extraction in duplicate?

Item7: Did the review authors provide a list of excluded studies and justify the exclusions?

Item8: Did the review authors describe the included studies in adequate detail?

Item9: Did the review authors use a satisfactory technique for assessing the risk of bias (RoB) in individual studies that were included in the review?

Item10: Did the review authors report on the sources of funding for the studies included in the review?

Item11: If meta-analysis was performed did the review authors use appropriate methods for statistical combination of results?

Item12: If meta-analysis was performed, did the review authors assess the potential impact of RoB in individual studies on the results of the meta-analysis or other evidence synthesis?

Item13: Did the review authors account for RoB in individual studies when interpreting/discussing the results of the review?

Item14: Did the review authors provide a satisfactory explanation for, and discussion of, any heterogeneity observed in the results of the review?

Item15: If they performed quantitative synthesis did the review authors carry out an adequate investigation of publication bias (small study bias) and discuss its likely impact on the results of the review?

Item16: Did the review authors report any potential sources of conflflict of interest, including any funding they received for conducting the review?

From:Shea BJ, Reeves BC, Wells G, Thuku M, Hamel C, Moran J, et al. AMSTAR 2: a critical appraisal tool for systematic reviews that include randomised or non-randomised studies of healthcare interventions, or both. *BMJ*. (2017) 358:j4008. doi:10.1136/bmj.j4008

3 Appendix C: Summary of Phase 2 domains, Phase 3, and Signaling Questions of Risk of Bias in Systematic reviews (ROBIS) .

	Phase 2				Phase 3
	**1. Study eligibility criteria**	**2. Identification and selection of studies**	**3. Data collection and study appraisal**	**4. Synthesis and findings**	**Risk of bias in the review**
Signaling questions	1.1 Did the review adhere to predefined objectives and eligibility criteria?	2.1 Did the search include an appropriate range of databases/ electronic sources for published and unpublished reports?	3.1. Were efforts made to minimize error in data collection?	4.1. Did the synthesis include all studies that it should?	A. Did the interpretation of findings address all of the concerns identified in domains 1 to 4?
	1.2 Were the eligibility criteria appropriate for the review question?	2.2 Were methods additional to database searching used to identify relevant reports?	3.2. Were sufficient study characteristics available for both review authors and readers to be able to interpret the results?	4.2. Were all predefined analyses reported or departures explained?	B. Was the relevance of identified studies to the review's research question appropriately considered?
	1.3 Were eligibility criteria unambiguous?	2.3 Were the terms and structure of the search strategy likely to retrieve as many eligible studies as possible?	3.3. Were all relevant study results collected for use in the synthesis?	4.3. Was the synthesis appropriate given the nature and similarity in the research questions, study designs, and outcomes across included studies?	C. Did the reviewers avoid emphasizing results on the basis of their statistical significance?
	1.4 Were all restrictions in eligibility criteria based on study characteristics appropriate?	2.4 Were restrictions based on date, publication format, or language appropriate?	3.4. Was risk of bias (or methodologic quality) formally assessed using appropriate criteria?	4.4. Was between-study variation minimal or addressed in the synthesis?	
	1.5 Were any restrictions in eligibility criteria based on sources of information appropriate?	2.5 Were efforts made to minimize error in selection of studies?	3.5. Were efforts made to minimize error in risk of bias assessment?	4.5. Were the findings robust, for example, as demonstrated through funnel plot or sensitivity analyses?	
				4.6. Were biases in primary studies minimal or addressed in the synthesis?	
Judgment	Concerns regarding specification of study eligibility criteria	Concerns regarding methods used to identify and/or select studies	Concerns regarding methods used to collect data and appraise studies	Concerns regarding the synthesis	Risk of bias in the review

From: Whiting P, Savović J, Higgins JP, Caldwell DM, Reeves BC, Shea B, et al. ROBIS: A new tool to assess risk of bias in systematic reviews was developed. *J Clin Epidemiol*. (2016) 69:225-34. doi:10.1016/j.jclinepi.2015.06.005

4 Appendix D: PRISMA 2020 Item Checklist

Section and topic	Item	Checklist item	Location
		Title	
Title	1	Identify the report as a systematic review.	
		Abstract	
Abstract	2	See the PRISMA 2020 for Abstracts checklist.	
		Introduction	
Rationale	3	Describe the rationale for the review in the context of existing knowledge.	
Objectives	4	Provide an explicit statement of the objective(s) or question(s) the review addresses.	
		Methods	
Eligibility criteria	5	Specify the inclusion and exclusion criteria for the review and how studies were grouped for the syntheses.	
Information sources	6	Specify all databases, registers, websites, organisations, reference lists and other sources searched or consulted to identify studies. Specify the date when each source was last searched or consulted.	
Search strategy	7	Present the full search strategies for all databases, registers and websites, including any filters and limits used.	
Selection process	8	Specify the methods used to decide whether a study met the inclusion criteria of the review, including how many reviewers screened each record and each report retrieved, whether they worked independently, and if applicable, details of automation tools used in the process.	
Data collection process	9	Specify the methods used to collect data from reports, including how many reviewers collected data from each report, whether they worked independently, any processes for obtaining or confirming data from study investigators, and if applicable, details of automation tools used in the process.	
Data items	10a	List and define all outcomes for which data were sought. Specify whether all results that were compatible with each outcome domain in each study were sought (e.g. for all measures, time points, analyses), and if not, the methods used to decide which results to collect.	
	10b	List and define all other variables for which data were sought (e.g. participant and intervention characteristics, funding sources).	

		Describe any assumptions made about any missing or unclear information.
Study risk of bias assessment	11	Specify the methods used to assess risk of bias in the included studies, including details of the tool(s) used, how many reviewers assessed each study and whether they worked independently, and if applicable, details of automation tools used in the process.
Effect measures	12	Specify for each outcome the effect measure(s) (e.g., risk ratio, mean difference) used in the synthesis or presentation of results.
Synthesis methods	13a	Describe the processes used to decide which studies were eligible for each synthesis (e.g., tabulating the study intervention characteristics and comparing against the planned groups for each synthesis (item #5)).
	13b	Describe any methods required to prepare the data for presentation or synthesis, such as handling of missing summary statistics, or data conversions.
	13c	Describe any methods used to tabulate or visually display results of individual studies and syntheses.
	13d	Describe any methods used to synthesise results and provide a rationale for the choice(s). If meta-analysis was performed, describe the model(s), method(s) to identify the presence and extent of statistical heterogeneity, and software package(s) used.
	13e	Describe any methods used to explore possible causes of heterogeneity among study results (e.g., subgroup analysis, metaregression).
	13f	Describe any sensitivity analyses conducted to assess robustness of the synthesised results.
Reporting bias assessment	14	Describe any methods used to assess risk of bias due to missing results in a synthesis (arising from reporting biases).
Certainty assessment	15	Describe any methods used to assess certainty (or confidence) in the body of evidence for an outcome.
Results		
Study selection	16a	Describe the results of the search and selection process, from the number of records identified in the search to the number of studies included in the review, ideally using a flow diagram.
	16b	Cite studies that might appear to meet the inclusion criteria, but which were excluded, and explain why they were excluded.
Study characteristics	17	Cite each included study and present its characteristics.
Risk of bias in studies	18	Present assessments of risk of bias for each included study.

Results of individual studies	19	For all outcomes, present, for each study: (a) summary statistics for each group (where appropriate) and (b) an effect estimate and its precision (e.g., confidence/credible interval), ideally using structured tables or plots.
Results of syntheses	20a	For each synthesis, briefly summarise the characteristics and risk of bias among contributing studies.
	20b	Present results of all statistical syntheses conducted. If meta-analysis was done, present for each the summary estimate and its precision (e.g., confidence/credible interval) and measures of statistical heterogeneity. If comparing groups, describe the direction of the effect.
	20c	Present results of all investigations of possible causes of heterogeneity among study results.
	20d	Present results of all sensitivity analyses conducted to assess the robustness of the synthesised results.
Reporting biases	21	Present assessments of risk of bias due to missing results (arising from reporting biases) for each synthesis assessed.
Certainty of evidence	22	Present assessments of certainty (or confidence) in the body of evidence for each outcome assessed.
**Discussion**		
Discussion	23a	Provide a general interpretation of the results in the context of other evidence.
	23b	Discuss any limitations of the evidence included in the review.
	23c	Discuss any limitations of the review processes used.
	23d	Discuss implications of the results for practice, policy, and future research.
**Other information**		
Registration and protocol	24a	Provide registration information for the review, including register name and registration number, or state that the review was not registered.
	24b	Indicate where the review protocol can be accessed, or state that a protocol was not prepared
	24c	Describe and explain any amendments to information provided at registration or in the protocol.
Support	25	Describe sources of financial or nonfinancial support for the review, and the role of the funders or sponsors in the review.
Competing interests	26	Declare any competing interests of review authors.
Availability of data, code, and other materials	27	Report which of the following are publicly available and where they can be found: template data collection forms; data extracted from included studies; data used for all analyses; analytic code; any other materials used in the review.

From: Page MJ, McKenzie JE, Bossuyt PM, Boutron I, Hoffmann TC, Mulrow CD, et al. The PRISMA 2020 statement: An updated guideline for reporting systematic reviews. *J Clin Epidemiol*. (2021) 134:178-89. doi:10.1016/j.jclinepi.2021.03.001

5 Appendix E: Quality of Evidence and Definitions for Grades of Recommendations, Assessment, Development and Evaluation (GRADE).

High	No or one non-critical weakness: the systematic review provides an accurate and comprehensive summary of the results of the available studies that address the question of interest.
High quality	Further research is very unlikely to change our confidence in the estimate of effect.
Moderate quality	Further research is likely to have an important impact on our confidence in the estimate of effect and may change the estimate.
Low quality	Further research is very likely to have an important impact on our confidence in the estimate of effect and is likely to change the estimate.
Critically low quality	Any estimate of effect is very uncertain.

From: Atkins D, Best D, Briss PA, Eccles M, Falck-Ytter Y, Flottorp S, et al. Grading quality of evidence and strength of recommendations. *BMJ*. (2004) 328:1490. doi:10.1136/bmj.328.7454.1490

6 Appendix F: The List of Excluded References.

	First author (year)	Title	Reason for exclusion
1	Shi (2021)	A comparison of the effects of Chinese non-pharmaceutical therapies for pain control in knee osteoarthritis: A protocol for a systematic review and network meta-analysis	Protocol
2	Pang (2022)	Effectiveness and safety of aromatherapy massage for knee osteoarthritis A protocol for systematic review and meta-analysis	Protocol
3	Xu (2022)	Effectiveness and safety of massage for chronic pain in patients with knee osteoarthritis: A protocol for systematic review and meta-analysis	Protocol
4	Qin (2020)	Effectiveness and safety of massage for knee osteoarthritis: A protocol for systematic review and meta-analysis	Protocol
5	Chang (2021)	The effects on pain and disability of traditional Chinese non-pharmacological therapy for knee osteoarthritis: A protocol for systematic review and meta-analysis	Protocol
6	Zheng (2022)	The effects on pain and quality of life of traditional Chinese manual therapy for knee osteoarthritis A protocol for systematic review and meta-analysis	Protocol
7	Yuan (2012)	A massage therapy on pain relief for knee osteoarthritis: A systematic review and meta-analysis	Insufficient data
8	Chen (2018)	Manual therapy for knee osteoarthritis pain: A systematic review and meta-analysis	Insufficient data
9	Bai (2018)	Massage combined with Chinese medicine external for knee osteoarthritis: A meta-analysis	Insufficient data

10	Ma (2019)	Meta-analysis of randomized controlled trials of Tuina in the treatment of knee osteoarthritis	Insufficient data
11	Weleslassie (2021)	Effectiveness of Mobilization with Movement on the Management of Knee Osteoarthritis: A Systematic Review of Randomized Controlled Trials	Narrative SR
12	Tsokanos (2021)	The Efficacy of Manual Therapy in Patients with Knee Osteoarthritis: A Systematic Review	Narrative SR
13	Xu (2015)	The effectiveness of manual therapy for relieving pain, stiffness and dysfunction in knee osteoarthritis: A systematic review and meta-analysis	Duplicate publication
14	Xing (2021)	Therapeutic massage for knee osteoarthritis: a systematic review and meta-analysis of randomized controlled trials	Duplicate publication
15	Li (2022)	Effectiveness of Maitland and Mulligan mobilization methods for adults with knee osteoarthritis: A systematic review and meta analysis	The control intervention is manual therapy
16	Salamh (2017)	Treatment effectiveness and fidelity of manual therapy to the knee: A systematic review and meta-analysis	SR/MA of non-RCT

References:

1. Shi X, Yu W, Wang D, Zhao Y, Deng X, Chen C, et al. A comparison of the effects of Chinese non-pharmaceutical therapies for pain control in knee osteoarthritis: A protocol for a systematic review and network meta-analysis. *Medicine (Baltimore)*. (2021) 100:e24501. doi:10.1097/MD.0000000000024501

2. Pang T, Liu C, Li J, Yao J, Li Z, Lei S,et al. Effectiveness and safety of aromatherapy massage for knee osteoarthritis: A protocol for systematic review and meta-analysis. *Medicine (Baltimore)*. (2022) 101:e29039. doi:10.1097/MD.0000000000029039

3. Xu J, Wu B, Xie S, Wu G, Zhang H, Fu Y, et al. Effectiveness and safety of massage for chronic pain in patients with knee osteoarthritis: A protocol for systematic review and meta-analysis. *Medicine (Baltimore)*. (2022) 101:e28533. doi:10.1097/MD.0000000000028533

4. Qin S, Chi Z, Xiao Y, Zhu D, Zhong G, Xu W, et al. Effectiveness and safety of massage for knee osteoarthritis: A protocol for systematic review and meta-analysis. *Medicine (Baltimore)*. (2020) 99:e22853. doi:10.1097/MD.0000000000022853

5. Chang W, Guo W, Wang R, Lin X, Sun S, Shi Y. The effects on pain and disability of traditional Chinese non-pharmacological therapy for knee osteoarthritis: A protocol for systematic review and meta-analysis. *Medicine (Baltimore)*. (2021) 100:e27005. doi:10.1097/MD.0000000000027005

6. Zheng Y, Ren J, Zhang S, Zhou X, He T, Kong L. The effects on pain and quality of life of traditional Chinese manual therapy for knee osteoarthritis: A protocol for systematic review and meta-analysis. *Medicine (Baltimore)*. (2022) 101:e28595. doi:10.1097/MD.0000000000028595

7. Yuan W, Bannuru RR, Kong L, Cheng Y, McAlindon T, Fang M, et, al. A massage therapy on pain relief for knee osteoarthritis: A systematic review and meta-analysis. *Osteoarthritis and Cartilage*. (2012) 20:S281. doi:10.1016/j.joca.2012.02.482

8. Chen B, Zhan H, Marszalek J, Chung M, Lin X, Bannuru R, et al. Manual therapy for knee osteoarthritis pain: A systematic review and meta-analysis. *Osteoarthritis and Cartilage*. (2018) 26:S318-19. doi:10.1016/j.joca.2018.02.636

9. Bai Y, Xie L, Ding Q, Xu Y, Wang D, Wang L. Meta-analysis of the efficacy of massage combined with external use of traditional Chinese medicine in the treatment of knee osteoarthritis. *Chinese Journal for Clinicians*. (2018) 46:1245-47. doi: 10.3969/j.issn.2095-8552.2018.10.036

10. Ma L, Liang S, Xie Y, Yang T. Meta analysis of randomized controlled trials of Tuina in the treatment of knee osteoarthritis. *Clinical Journal of Chinese Medicine*. (2019) 11:100-03. doi:10.3969/j.issn.1674-7860.2019.06.044

11. Weleslassie GG, Temesgen MH, Alamer A, Tsegay GS, Hailemariam TT, Melese H. Effectiveness of Mobilization with Movement on the Management of Knee Osteoarthritis: A Systematic Review of Randomized Controlled Trials. *Pain Res Manag*. (2021) 2021:8815682. doi:10.1155/2021/8815682

12. Tsokanos A, Livieratou E, Billis E, Tsekoura M, Tatsios P, Tsepis E, et al. The Efficacy of Manual Therapy in Patients with Knee Osteoarthritis: A Systematic Review. *Medicina (Kaunas)*. (2021) 57:696. doi:10.3390/medicina57070696

13. Xu Q, Pang J, Zheng Y, Zhan H, Cao Y, Ding C. The effectiveness of manual therapy for relieving pain, stiffness and dysfunction in knee osteoarthritis: A systematic review and meta-analysis. *Osteoarthritis and Cartilage*. (2015) 23: A387. doi: 10.1016/j.joca.2015.02.715

14. Xing H, Shen J, Gong L, Yao F, Li J, Shao S, et al. Therapeutic massage for knee osteoarthritis: a systematic review and meta-analysis of randomized controlled trials. *J Acupunct Tuina Sci*. (2021) 19:354-63. doi:10.1007/s11726-021-1266-4

15. Li L, Hu X, Di Y, Jiao W. Effectiveness of Maitland and Mulligan mobilization methods for adults with knee osteoarthritis: A systematic review and meta-analysis. *World J Clin Cases*. (2022) 10:954-65. doi:10.12998/wjcc.v10.i3.954

16. Salamh P, Cook C, Reiman MP, Sheets C. Treatment effectiveness and fidelity of manual therapy to the knee: A systematic review and meta-analysis. *Musculoskeletal Care*. (2017) 15:238-48. doi:10.1002/msc.1166
